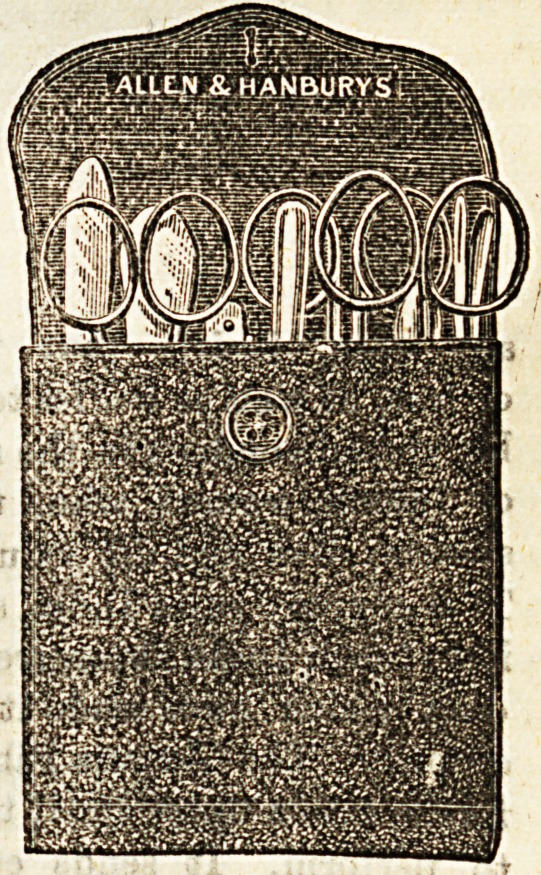# New Drugs, Appliances, and Things Medical

**Published:** 1891-09-26

**Authors:** 


### NEW DRUGS, APPLIANCES, AND THINGS MEDICAL.

[All preparations, appliances, novelties, etc., of which a notice is desired, should be sent for The Editor, to care of The Manager, 140, Strand, London, W.C.]

### NEW NURSES' CASE.

Messrs. Allen and Hanbury have endeavoured to execute the difficult task of supplying a new form of instrument case for nurses. Their efforts have not been in vain. The article in question is a combined chatelaine and pocket-case in durable leather. It contains scissors, spatula, thermometer, knife, probes, &c., nine instruments in all. It differs in construction from cases in common use, in being provided with a pocket for leaflets, match-box, and measure, and is arranged as a hanging case for the nurse's bedside. With all this combination of usefulness, the case is little larger than those of a less comprehensive nature.

## Figures and Tables

**Figure f1:**
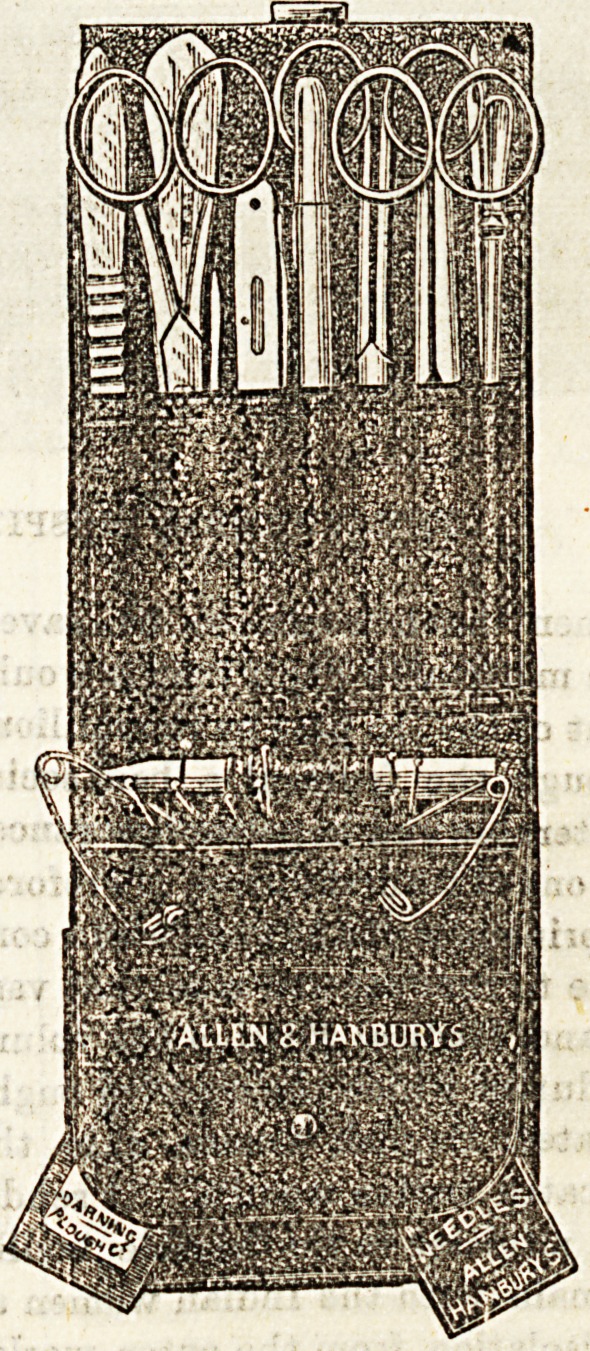


**Figure f2:**